# Mental Health, Substance Use, and Suicidal Ideation During the COVID-19 Pandemic — United States, June 24–30, 2020

**DOI:** 10.15585/mmwr.mm6932a1

**Published:** 2020-08-14

**Authors:** Mark É. Czeisler, Rashon I. Lane, Emiko Petrosky, Joshua F. Wiley, Aleta Christensen, Rashid Njai, Matthew D. Weaver, Rebecca Robbins, Elise R. Facer-Childs, Laura K. Barger, Charles A. Czeisler, Mark E. Howard, Shantha M.W. Rajaratnam

**Affiliations:** ^1^Turner Institute for Brain and Mental Health, Monash University, Melbourne, Australia; ^2^Austin Health, Melbourne, Australia; ^3^CDC COVID-19 Response Team; ^4^Brigham and Women’s Hospital, Boston, Massachusetts; ^5^Harvard Medical School, Boston, Massachusetts; ^6^University of Melbourne, Melbourne, Australia.

The coronavirus disease 2019 (COVID-19) pandemic has been associated with mental health challenges related to the morbidity and mortality caused by the disease and to mitigation activities, including the impact of physical distancing and stay-at-home orders.* Symptoms of anxiety disorder and depressive disorder increased considerably in the United States during April–June of 2020, compared with the same period in 2019 (*1*,*2*). To assess mental health, substance use, and suicidal ideation during the pandemic, representative panel surveys were conducted among adults aged ≥18 years across the United States during June 24–30, 2020. Overall, 40.9% of respondents reported at least one adverse mental or behavioral health condition, including symptoms of anxiety disorder or depressive disorder (30.9%), symptoms of a trauma- and stressor-related disorder (TSRD) related to the pandemic^†^ (26.3%), and having started or increased substance use to cope with stress or emotions related to COVID-19 (13.3%). The percentage of respondents who reported having seriously considered suicide in the 30 days before completing the survey (10.7%) was significantly higher among respondents aged 18–24 years (25.5%), minority racial/ethnic groups (Hispanic respondents [18.6%], non-Hispanic black [black] respondents [15.1%]), self-reported unpaid caregivers for adults^§^ (30.7%), and essential workers^¶^ (21.7%). Community-level intervention and prevention efforts, including health communication strategies, designed to reach these groups could help address various mental health conditions associated with the COVID-19 pandemic.

During June 24–30, 2020, a total of 5,412 (54.7%) of 9,896 eligible invited adults** completed web-based surveys^††^ administered by Qualtrics.^§§^ The Monash University Human Research Ethics Committee of Monash University (Melbourne, Australia) reviewed and approved the study protocol on human subjects research. Respondents were informed of the study purposes and provided electronic consent before commencement, and investigators received anonymized responses. Participants included 3,683 (68.1%) first-time respondents and 1,729 (31.9%) respondents who had completed a related survey during April 2–8, May 5–12, 2020, or both intervals; 1,497 (27.7%) respondents participated during all three intervals (*2*,*3*). Quota sampling and survey weighting were employed to improve cohort representativeness of the U.S. population by gender, age, and race/ethnicity.^¶¶^ Symptoms of anxiety disorder and depressive disorder were assessed using the four-item Patient Health Questionnaire*** (*4*), and symptoms of a COVID-19–related TSRD were assessed using the six-item Impact of Event Scale^†††^ (*5*). Respondents also reported whether they had started or increased substance use to cope with stress or emotions related to COVID-19 or seriously considered suicide in the 30 days preceding the survey.^§§§^

Analyses were stratified by gender, age, race/ethnicity, employment status, essential worker status, unpaid adult caregiver status, rural-urban residence classification,^¶¶¶^ whether the respondent knew someone who had positive test results for SARS-CoV-2, the virus that causes COVID-19, or who had died from COVID-19, and whether the respondent was receiving treatment for diagnosed anxiety, depression, or posttraumatic stress disorder (PTSD) at the time of the survey. Comparisons within subgroups were evaluated using Poisson regressions with robust standard errors to calculate prevalence ratios, 95% confidence intervals (CIs), and p-values to evaluate statistical significance (α = 0.005 to account for multiple comparisons). Among the 1,497 respondents who completed all three surveys, longitudinal analyses of the odds of incidence**** of symptoms of adverse mental or behavioral health conditions by essential worker and unpaid adult caregiver status were conducted on unweighted responses using logistic regressions to calculate unadjusted and adjusted^††††^ odds ratios (ORs), 95% CI, and p-values (α = 0.05). The statsmodels package in Python (version 3.7.8; Python Software Foundation) was used to conduct all analyses.

Overall, 40.9% of 5,470 respondents who completed surveys during June reported an adverse mental or behavioral health condition, including those who reported symptoms of anxiety disorder or depressive disorder (30.9%), those with TSRD symptoms related to COVID-19 (26.3%), those who reported having started or increased substance use to cope with stress or emotions related to COVID-19 (13.3%), and those who reported having seriously considered suicide in the preceding 30 days (10.7%) (Table 1). At least one adverse mental or behavioral health symptom was reported by more than one half of respondents who were aged 18–24 years (74.9%) and 25–44 years (51.9%), of Hispanic ethnicity (52.1%), and who held less than a high school diploma (66.2%), as well as those who were essential workers (54.0%), unpaid caregivers for adults (66.6%), and who reported treatment for diagnosed anxiety (72.7%), depression (68.8%), or PTSD (88.0%) at the time of the survey.

**TABLE 1:** Respondent characteristics and prevalence of adverse mental health outcomes, increased substance use to cope with stress or emotions related to COVID-19 pandemic, and suicidal ideation — United States, June 24–30, 2020

Characteristic	All respondents who completed surveys during June 24–30, 2020 weighted* no. (%)	Weighted %*						
		Conditions				Started or increased substance use to cope with pandemic-related stress or emotions^¶^	Seriously considered suicide in past 30 days	≥1 adverse mental or behavioral health symptom
		Anxiety disorder^†^	Depressive disorder^†^	Anxiety or depressive disorder^†^	COVID-19–related TSRD^§^			
**All respondents**	**5,470 (100)**	**25.5**	**24.3**	**30.9**	**26.3**	**13.3**	**10.7**	**40.9**
**Gender**								
Female	2,784 (50.9)	26.3	23.9	31.5	24.7	12.2	8.9	41.4
Male	2,676 (48.9)	24.7	24.8	30.4	27.9	14.4	12.6	40.5
Other	10 (0.2)	20.0	30.0	30.0	30.0	10.0	0.0	30.0
**Age group (yrs)**								
18–24	731 (13.4)	49.1	52.3	62.9	46.0	24.7	25.5	74.9
25–44	1,911 (34.9)	35.3	32.5	40.4	36.0	19.5	16.0	51.9
45–64	1,895 (34.6)	16.1	14.4	20.3	17.2	7.7	3.8	29.5
≥65	933 (17.1)	6.2	5.8	8.1	9.2	3.0	2.0	15.1
**Race/Ethnicity**								
White, non-Hispanic	3,453 (63.1)	24.0	22.9	29.2	23.3	10.6	7.9	37.8
Black, non-Hispanic	663 (12.1)	23.4	24.6	30.2	30.4	18.4	15.1	44.2
Asian, non-Hispanic	256 (4.7)	14.1	14.2	18.0	22.1	6.7	6.6	31.9
Other race or multiple races, non-Hispanic**	164 (3.0)	27.8	29.3	33.2	28.3	11.0	9.8	43.8
Hispanic, any race(s)	885 (16.2)	35.5	31.3	40.8	35.1	21.9	18.6	52.1
Unknown	50 (0.9)	38.0	34.0	44.0	34.0	18.0	26.0	48.0
**2019 Household income (USD)**								
<25,000	741 (13.6)	30.6	30.8	36.6	29.9	12.5	9.9	45.4
25,000–49,999	1,123 (20.5)	26.0	25.6	33.2	27.2	13.5	10.1	43.9
50,999–99,999	1,775 (32.5)	27.1	24.8	31.6	26.4	12.6	11.4	40.3
100,999–199,999	1,301 (23.8)	23.1	20.8	27.7	24.2	15.5	11.7	37.8
≥200,000	282 (5.2)	17.4	17.0	20.6	23.1	14.8	11.6	35.1
Unknown	247 (4.5)	19.6	23.1	27.2	24.9	6.2	3.9	41.5
**Education**								
Less than high school diploma	78 (1.4)	44.5	51.4	57.5	44.5	22.1	30.0	66.2
High school diploma	943 (17.2)	31.5	32.8	38.4	32.1	15.3	13.1	48.0
Some college	1,455 (26.6)	25.2	23.4	31.7	22.8	10.9	8.6	39.9
Bachelor's degree	1,888 (34.5)	24.7	22.5	28.7	26.4	14.2	10.7	40.6
Professional degree	1,074 (19.6)	20.9	19.5	25.4	24.5	12.6	10.0	35.2
Unknown	33 (0.6)	25.2	23.2	28.2	23.2	10.5	5.5	28.2
**Employment status^††^**								
Employed	3,431 (62.7)	30.1	29.1	36.4	32.1	17.9	15.0	47.8
Essential	1,785 (32.6)	35.5	33.6	42.4	38.5	24.7	21.7	54.0
Nonessential	1,646 (30.1)	24.1	24.1	29.9	25.2	10.5	7.8	41.0
Unemployed	761 (13.9)	32.0	29.4	37.8	25.0	7.7	4.7	45.9
Retired	1,278 (23.4)	9.6	8.7	12.1	11.3	4.2	2.5	19.6
**Unpaid adult caregiver status^§§^**								
Yes	1,435 (26.2)	47.6	45.2	56.1	48.4	32.9	30.7	66.6
No	4,035 (73.8)	17.7	16.9	22.0	18.4	6.3	3.6	31.8
**Region** ^¶¶^								
Northeast	1,193 (21.8)	23.9	23.9	29.9	22.8	12.8	10.2	37.1
Midwest	1,015 (18.6)	22.7	21.1	27.5	24.4	9.0	7.5	36.1
South	1,921 (35.1)	27.9	26.5	33.4	29.1	15.4	12.5	44.4
West	1,340 (24.5)	25.8	24.2	30.9	26.7	14.0	10.9	43.0
Rural-urban classification***								
Rural	599 (10.9)	26.0	22.5	29.3	25.4	11.5	10.2	38.3
Urban	4,871 (89.1)	25.5	24.6	31.1	26.4	13.5	10.7	41.2
**Know someone who had positive test results for SARS-CoV-2**								
Yes	1,109 (20.3)	23.8	21.9	29.6	21.5	12.9	7.5	39.2
No	4,361 (79.7)	26.0	25.0	31.3	27.5	13.4	11.5	41.3
**Knew someone who died from COVID-19**								
Yes	428 (7.8)	25.8	20.6	30.6	28.1	11.3	7.6	40.1
No	5,042 (92.2)	25.5	24.7	31.0.	26.1	13.4	10.9	41.0
**Receiving treatment for previously diagnosed condition**								
**Anxiety**								
Yes	536 (9.8)	59.6	52.0	66.0	51.9	26.6	23.6	72.7
No	4,934 (90.2)	21.8	21.3	27.1	23.5	11.8	9.3	37.5
**Depression**								
Yes	540 (9.9)	52.5	50.6	60.8	45.5	25.2	22.1	68.8
No	4,930 (90.1)	22.6	21.5	27.7	24.2	12.0	9.4	37.9
**Posttraumatic stress disorder**								
Yes	251 (4.6)	72.3	69.1	78.7	69.4	43.8	44.8	88.0
No	5,219 (95.4)	23.3	22.2	28.6	24.2	11.8	9.0	38.7

**Abbreviations**: COVID-19 = coronavirus disease 2019; TSRD = trauma- and stressor-related disorder.

* Survey weighting was employed to improve the cross-sectional June cohort representativeness of the U.S. population by gender, age, and race/ethnicity according to the 2010 U.S. Census with respondents in which gender, age, and race/ethnicity were reported. Respondents who reported a gender of “Other” or who did not report race/ethnicity were assigned a weight of one.

^†^ Symptoms of anxiety disorder and depressive disorder were assessed via the four-item Patient Health Questionnaire (PHQ-4). Those who scored ≥3 out of 6 on the Generalized Anxiety Disorder (GAD-2) and Patient Health Questionnaire (PHQ-2) subscales were considered symptomatic for each disorder, respectively.

^§^ Disorders classified as TSRDs in the *Diagnostic and Statistical Manual of Mental Disorders* (DSM–5) include posttraumatic stress disorder (PTSD), acute stress disorder (ASD), and adjustment disorders (ADs), among others. Symptoms of a TSRD precipitated by the COVID-19 pandemic were assessed via the six-item Impact of Event Scale (IES-6) to screen for overlapping symptoms of PTSD, ASD, and ADs. For this survey, the COVID-19 pandemic was specified as the traumatic exposure to record peri- and posttraumatic symptoms associated with the range of stressors introduced by the COVID-19 pandemic. Those who scored ≥1.75 out of 4 were considered symptomatic.

^¶^ 104 respondents selected “Prefer not to answer.”

** The Other race or multiple races, non-Hispanic category includes respondents who identified as not being Hispanic and as more than one race or as American Indian or Alaska Native, Native Hawaiian or Pacific Islander, or “Other.”

^††^ Essential worker status was self-reported. The comparison was between employed respondents (n = 3,431) who identified as essential vs. nonessential. For this analysis, students who were not separately employed as essential workers were considered nonessential workers.

^§§^ Unpaid adult caregiver status was self-reported. The definition of an unpaid caregiver for adults was a person who had provided unpaid care to a relative or friend aged ≥18 years to help them take care of themselves at any time in the last 3 months. Examples provided included helping with personal needs, household chores, health care tasks, managing a person’s finances, taking them to a doctor’s appointment, arranging for outside services, and visiting regularly to see how they are doing.

^¶¶^ Region classification was determined by using the U.S. Census Bureau’s Census Regions and Divisions of the United States. https://www2.census.gov/geo/pdfs/maps-data/maps/reference/us_regdiv.pdf.

*** Rural-urban classification was determined by using self-reported ZIP codes according to the Federal Office of Rural Health Policy definition of rurality. https://www.hrsa.gov/rural-health/about-us/definition/datafiles.html.

Prevalences of symptoms of adverse mental or behavioral health conditions varied significantly among subgroups (Table 2). Suicidal ideation was more prevalent among males than among females. Symptoms of anxiety disorder or depressive disorder, COVID-19–related TSRD, initiation of or increase in substance use to cope with COVID-19–associated stress, and serious suicidal ideation in the previous 30 days were most commonly reported by persons aged 18–24 years; prevalence decreased progressively with age. Hispanic respondents reported higher prevalences of symptoms of anxiety disorder or depressive disorder, COVID-19–related TSRD, increased substance use, and suicidal ideation than did non-Hispanic whites (whites) or non-Hispanic Asian (Asian) respondents. Black respondents reported increased substance use and past 30-day serious consideration of suicide in the previous 30 days more commonly than did white and Asian respondents. Respondents who reported treatment for diagnosed anxiety, depression, or PTSD at the time of the survey reported higher prevalences of symptoms of adverse mental and behavioral health conditions compared with those who did not. Symptoms of a COVID-19–related TSRD, increased substance use, and suicidal ideation were more prevalent among employed than unemployed respondents, and among essential workers than nonessential workers. Adverse conditions also were more prevalent among unpaid caregivers for adults than among those who were not, with particularly large differences in increased substance use (32.9% versus 6.3%) and suicidal ideation (30.7% versus 3.6%) in this group.

**TABLE 2:** Comparison of symptoms of adverse mental health outcomes among all respondents who completed surveys (N = 5,470), by respondent characteristic* — United States, June 24–30, 2020

Characteristic	Prevalence ratio^¶^ (95% CI^¶^)			
	Symptoms of anxiety disorder or depressive disorder^†^	Symptoms of a TSRD related to COVID-19^§^	Started or increased substance use to cope with stress or emotions related to COVID-19	Serious consideration of suicide in past 30 days
**Gender**				
Female vs. male	1.04 (0.96–1.12)	0.88 (0.81–0.97)	0.85 (0.75–0.98)	0.70 (0.60–0.82)**
**Age group (yrs)**				
18–24 vs. 25–44	1.56 (1.44–1.68)**	1.28 (1.16–1.41)**	1.31 (1.12–1.53)**	1.59 (1.35–1.87)**
18–24 vs. 45–64	3.10 (2.79–3.44)**	2.67 (2.35–3.03)**	3.35 (2.75–4.10)**	6.66 (5.15–8.61)**
18–24 vs. ≥65	7.73 (6.19–9.66)**	5.01 (4.04–6.22)**	8.77 (5.95–12.93)**	12.51 (7.88–19.86)**
25–44 vs. 45–64	1.99 (1.79–2.21)**	2.09 (1.86–2.35)**	2.56 (2.14–3.07)**	4.18 (3.26–5.36)**
25–44 vs. ≥65	4.96 (3.97–6.20)**	3.93 (3.18–4.85)**	6.70 (4.59–9.78)**	7.86 (4.98–12.41)**
45–64 vs. ≥65	2.49 (1.98–3.15)**	1.88 (1.50–2.35)**	2.62 (1.76–3.9)**	1.88 (1.14–3.10)
**Race/Ethnicity^††^**				
Hispanic vs. non-Hispanic black	1.35 (1.18–1.56)**	1.15 (1.00–1.33)	1.19 (0.97–1.46)	1.23 (0.98–1.55)
Hispanic vs. non-Hispanic Asian	2.27 (1.73–2.98)**	1.59 (1.24–2.04)**	3.29 (2.05–5.28)**	2.82 (1.74–4.57)**
Hispanic vs. non-Hispanic other race or multiple races	1.23 (0.98–1.55)	1.24 (0.96–1.61)	1.99 (1.27–3.13)**	1.89 (1.16–3.06)
Hispanic vs. non-Hispanic white	1.40 (1.27–1.54)**	1.50 (1.35–1.68)**	2.09 (1.79–2.45)**	2.35 (1.96–2.80)**
Non-Hispanic black vs. non-Hispanic Asian	1.68 (1.26–2.23)**	1.38 (1.07–1.78)	2.75 (1.70–4.47)**	2.29 (1.39–3.76)**
Non-Hispanic black vs. non-Hispanic other race or multiple races	0.91 (0.71–1.16)	1.08 (0.82–1.41)	1.67 (1.05–2.65)	1.53 (0.93–2.52)
Non-Hispanic black vs. non-Hispanic white	1.03 (0.91–1.17)	1.30 (1.14–1.48)**	1.75 (1.45–2.11)**	1.90 (1.54–2.36)**
Non-Hispanic Asian vs. non-Hispanic other race or multiple races	0.54 (0.39–0.76)**	0.78 (0.56–1.09)	0.61 (0.32–1.14)	0.67 (0.35–1.29)
Non-Hispanic Asian vs. non-Hispanic white	0.62 (0.47–0.80)**	0.95 (0.74–1.20)	0.64 (0.40–1.02)	0.83 (0.52–1.34)
Non-Hispanic other race or multiple races vs. non-Hispanic white	1.14 (0.91–1.42)	1.21 (0.94–1.56)	1.05 (0.67–1.64)	1.24 (0.77–2)
**Employment status**				
Employed vs. unemployed	0.96 (0.87–1.07)	1.28 (1.12–1.46)**	2.30 (1.78–2.98)**	3.21 (2.31–4.47)**
Employed vs. retired	3.01 (2.58–3.51)**	2.84 (2.42–3.34)**	4.30 (3.28–5.63)**	5.97 (4.20–8.47)**
Unemployed vs. retired	3.12 (2.63–3.71)**	2.21 (1.82–2.69)**	1.87 (1.30–2.67)**	1.86 (1.16–2.96)
**Essential vs. nonessential worker^§§^**	1.42 (1.30–1.56)**	1.52 (1.38–1.69)**	2.36 (2.00–2.77)**	2.76 (2.29–3.33)**
**Unpaid caregiver for adults vs. not^¶¶`^**	2.55 (2.37–2.75)**	2.63 (2.42–2.86)**	5.28 (4.59–6.07)**	8.64 (7.23–10.33)**
Rural vs. urban residence***	0.94 (0.82–1.07)	0.96 (0.83–1.11)	0.84 (0.67–1.06)	0.95 (0.74–1.22)
**Knows someone with positive SARS-CoV-2 test result vs. not**	0.95 (0.86–1.05)	0.78 (0.69–0.88)**	0.96 (0.81–1.14)	0.65 (0.52–0.81)**
**Knew someone who died from COVID-19 vs. not**	0.99 (0.85–1.15)	1.08 (0.92–1.26)	0.84 (0.64–1.11)	0.69 (0.49–0.97)
**Receiving treatment for anxiety vs. not**	2.43 (2.26–2.63)**	2.21 (2.01–2.43)**	2.27 (1.94–2.66)**	2.54 (2.13–3.03)**
**Receiving treatment for depression vs. not**	2.20 (2.03–2.39)**	1.88 (1.70–2.09)**	2.13 (1.81–2.51)**	2.35 (1.96–2.82)**
**Receiving treatment for PTSD vs. not**	2.75 (2.55–2.97)**	2.87 (2.61–3.16)**	3.78 (3.23–4.42)**	4.95 (4.21–5.83)**

**Abbreviations**: CI = confidence interval; COVID-19 = coronavirus disease 2019; PTSD = posttraumatic stress disorder; TSRD = trauma- and stressor-related disorder.

* Number of respondents for characteristics: gender (female = 2,784, male = 2,676), age group in years (18–24 = 731; 25–44 = 1,911; 45–64 = 1,895; ≥65 = 933), race/ethnicity (non-Hispanic white = 3453, non-Hispanic black = 663, non-Hispanic Asian = 256, non-Hispanic other race or multiple races = 164, Hispanic = 885).

^†^ Symptoms of anxiety disorder and depressive disorder were assessed via the four-item Patient Health Questionnaire (PHQ-4). Those who scored ≥3 out of 6 on the Generalized Anxiety Disorder (GAD-2) and Patient Health Questionnaire (PHQ-2) subscales were considered to have symptoms of these disorders.

^§^ Disorders classified as TSRDs in the *Diagnostic and Statistical Manual of Mental Disorders* (DSM–5) include PTSD, acute stress disorder (ASD), and adjustment disorders (ADs), among others. Symptoms of a TSRD precipitated by the COVID-19 pandemic were assessed via the six-item Impact of Event Scale (IES-6) to screen for overlapping symptoms of PTSD, ASD, and ADs. For this survey, the COVID-19 pandemic was specified as the traumatic exposure to record peri- and posttraumatic symptoms associated with the range of stressors introduced by the COVID-19 pandemic. Persons who scored ≥1.75 out of 4 were considered to be symptomatic.

^¶^ Comparisons within subgroups were evaluated on weighted responses via Poisson regressions used to calculate a prevalence ratio, 95% CI, and p-value (not shown). Statistical significance was evaluated at a threshold of *α* = 0.005 to account for multiple comparisons. In the calculation of prevalence ratios for started or increased substance use, respondents who selected “Prefer not to answer” (n = 104) were excluded.

** P-value is statistically significant (p<0.005).

^††^ Respondents identified as a single race unless otherwise specified. The non-Hispanic, other race or multiple races category includes respondents who identified as not Hispanic and as more than one race or as American Indian or Alaska Native, Native Hawaiian or Pacific Islander, or ‘Other’.

^§§^ Essential worker status was self-reported. The comparison was between employed respondents (n = 3,431) who identified as essential vs. nonessential. For this analysis, students who were not separately employed as essential workers were considered nonessential workers.

^¶¶^ Unpaid adult caregiver status was self-reported. The definition of an unpaid caregiver for adults was having provided unpaid care to a relative or friend aged ≥18 years to help them take care of themselves at any time in the last 3 months. Examples provided included helping with personal needs, household chores, health care tasks, managing a person’s finances, taking them to a doctor’s appointment, arranging for outside services, and visiting regularly to see how they are doing.

*** Rural-urban classification was determined by using self-reported ZIP codes according to the Federal Office of Rural Health Policy definition of rurality. https://www.hrsa.gov/rural-health/about-us/definition/datafiles.html.

Longitudinal analysis of responses of 1,497 persons who completed all three surveys revealed that unpaid caregivers for adults had a significantly higher odds of incidence of adverse mental health conditions compared with others (Table 3). Among those who did not report having started or increased substance use to cope with stress or emotions related to COVID-19 in May, unpaid caregivers for adults had 3.33 times the odds of reporting this behavior in June (adjusted OR 95% CI = 1.75–6.31; p<0.001). Similarly, among those who did not report having seriously considered suicide in the previous 30 days in May, unpaid caregivers for adults had 3.03 times the odds of reporting suicidal ideation in June (adjusted OR 95% CI = 1.20–7.63; p = 0.019).

**TABLE 3:** Odds of incidence* of symptoms of adverse mental health, substance use to cope with stress or emotions related to COVID–19 pandemic, and suicidal ideation in the third survey wave, by essential worker status and unpaid adult caregiver status among respondents who completed monthly surveys from April through June (N = 1,497) — United States, April 2–8, May 5–12, and June 24–30, 2020

Symptom or behavior	Essential worker^†^ vs. all other employment statuses (nonessential worker, unemployed, retired)				Unpaid caregiver for adults^§^ vs. not unpaid caregiver			
	Unadjusted		Adjusted^¶^		Unadjusted		Adjusted**	
	OR (95% CI)^††^	p-value^††^	OR (95% CI)^††^	p-value^††^	OR (95% CI)^††^	p-value^††^	OR (95% CI)^††^	p-value^††^
**Symptoms of anxiety disorder^§§^**	1.92 (1.29–2.87)	0.001	1.63 (0.99–2.69)	0.056	1.97 (1.25–3.11)	0.004	1.81 (1.14–2.87)	0.012
**Symptoms of depressive disorder^§§^**	1.49 (1.00–2.22)	0.052	1.13 (0.70–1.82)	0.606	2.29 (1.50–3.50)	<0.001	2.22 (1.45–3.41)	<0.001
**Symptoms of anxiety disorder or depressive disorder^§§^**	1.67 (1.14–2.46)	0.008	1.26 (0.79–2.00)	0.326	1.84 (1.19–2.85)	0.006	1.73 (1.11–2.70)	0.015
**Symptoms of a TSRD related to** **COVID–19^¶¶^**	1.55 (0.86–2.81)	0.146	1.27 (0.63–2.56)	0.512	1.88 (0.99–3.56)	0.054	1.79 (0.94–3.42)	0.076
**Started or increased substance use to cope with stress or emotions related to COVID–19**	2.36 (1.26–4.42)	0.007	2.04 (0.92–4.48)	0.078	3.51 (1.86–6.61)	<0.001	3.33 (1.75–6.31)	<0.001
**Serious consideration of suicide in previous 30 days**	0.93 (0.31–2.78)	0.895	0.53 (0.16–1.70)	0.285	3.00 (1.20–7.52)	0.019	3.03 (1.20–7.63)	0.019

**Abbreviations**: CI = confidence interval, COVID–19 = coronavirus disease 2019, OR = odds ratio, TSRD = trauma– and stressor–related disorder.

* For outcomes assessed via the four-item Patient Health Questionnaire (PHQ–4), odds of incidence were marked by the presence of symptoms during May 5–12 or June 24–30, 2020, after the absence of symptoms during April 2–8, 2020. Respondent pools for prospective analysis of odds of incidence (did not screen positive for symptoms during April 2–8): anxiety disorder (n = 1,236), depressive disorder (n = 1,301) and anxiety disorder or depressive disorder (n = 1,190). For symptoms of a TSRD precipitated by COVID–19, started or increased substance use to cope with stress or emotions related to COVID–19, and serious suicidal ideation in the previous 30 days, odds of incidence were marked by the presence of an outcome during June 24–30, 2020, after the absence of that outcome during May 5–12, 2020. Respondent pools for prospective analysis of odds of incidence (did not report symptoms or behavior during May 5–12): symptoms of a TSRD (n = 1,206), started or increased substance use (n = 1,408), and suicidal ideation (n = 1,456).

^†^ Essential worker status was self–reported. For Table 3, essential worker status was determined by identification as an essential worker during the June 24–30 survey. Essential workers were compared with all other respondents, not just employed respondents (i.e., essential workers vs. all other employment statuses (nonessential worker, unemployed, and retired), not essential vs. nonessential workers).

^§^ Unpaid adult caregiver status was self–reported. The definition of an unpaid caregiver for adults was having provided unpaid care to a relative or friend 18 years or older to help them take care of themselves at any time in the last 3 months. Examples provided included helping with personal needs, household chores, health care tasks, managing a person’s finances, taking them to a doctor’s appointment, arranging for outside services, and visiting regularly to see how they are doing.

^¶^ Adjusted for gender, employment status, and unpaid adult caregiver status.

** Adjusted for gender, employment status, and essential worker status.

^††^ Respondents who completed surveys from all three waves (April, May, June) were eligible to be included in an unweighted longitudinal analysis. Comparisons within subgroups were evaluated via logit–linked Binomial regressions used to calculate unadjusted and adjusted odds ratios, 95% confidence intervals, and p–values. Statistical significance was evaluated at a threshold of α = 0.05. In the calculation of odds ratios for started or increased substance use, respondents who selected “Prefer not to answer” (n = 11) were excluded.

**^§§^** Symptoms of anxiety disorder and depressive disorder were assessed via the PHQ–4. Those who scored ≥3 out of 6 on the two–item Generalized Anxiety Disorder (GAD–2) and two-item Patient Health Questionnaire (PHQ–2) subscales were considered symptomatic for each disorder, respectively.

^¶¶^ Disorders classified as TSRDs in the *Diagnostic and Statistical Manual of Mental Disorders* (DSM–5) include posttraumatic stress disorder (PTSD), acute stress disorder (ASD), and adjustment disorders (ADs), among others. Symptoms of a TSRD precipitated by the COVID–19 pandemic were assessed via the six–item Impact of Event Scale (IES–6) to screen for overlapping symptoms of PTSD, ASD, and ADs. For this survey, the COVID–19 pandemic was specified as the traumatic exposure to record peri– and posttraumatic symptoms associated with the range of potential stressors introduced by the COVID–19 pandemic. Those who scored ≥1.75 out of 4 were considered symptomatic.

## Discussion

Elevated levels of adverse mental health conditions, substance use, and suicidal ideation were reported by adults in the United States in June 2020. The prevalence of symptoms of anxiety disorder was approximately three times those reported in the second quarter of 2019 (25.5% versus 8.1%), and prevalence of depressive disorder was approximately four times that reported in the second quarter of 2019 (24.3% versus 6.5%) (*2*). However, given the methodological differences and potential unknown biases in survey designs, this analysis might not be directly comparable with data reported on anxiety and depression disorders in 2019 (*2*). Approximately one quarter of respondents reported symptoms of a TSRD related to the pandemic, and approximately one in 10 reported that they started or increased substance use because of COVID-19. Suicidal ideation was also elevated; approximately twice as many respondents reported serious consideration of suicide in the previous 30 days than did adults in the United States in 2018, referring to the previous 12 months (10.7% versus 4.3%) (*6*).

Mental health conditions are disproportionately affecting specific populations, especially young adults, Hispanic persons, black persons, essential workers, unpaid caregivers for adults, and those receiving treatment for preexisting psychiatric conditions. Unpaid caregivers for adults, many of whom are currently providing critical aid to persons at increased risk for severe illness from COVID-19, had a higher incidence of adverse mental and behavioral health conditions compared with others. Although unpaid caregivers of children were not evaluated in this study, approximately 39% of unpaid caregivers for adults shared a household with children (compared with 27% of other respondents). Caregiver workload, especially in multigenerational caregivers, should be considered for future assessment of mental health, given the findings of this report and hardships potentially faced by caregivers.

The findings in this report are subject to at least four limitations. First, a diagnostic evaluation for anxiety disorder or depressive disorder was not conducted; however, clinically validated screening instruments were used to assess symptoms. Second, the trauma- and stressor-related symptoms assessed were common to multiple TSRDs, precluding distinction among them; however, the findings highlight the importance of including COVID-19–specific trauma measures to gain insights into peri- and posttraumatic impacts of the COVID-19 pandemic (*7*). Third, substance use behavior was self-reported; therefore, responses might be subject to recall, response, and social desirability biases. Finally, given that the web-based survey might not be fully representative of the United States population, findings might have limited generalizability. However, standardized quality and data inclusion screening procedures, including algorithmic analysis of click-through behavior, removal of duplicate responses and scrubbing methods for web-based panel quality were applied. Further the prevalence of symptoms of anxiety disorder and depressive disorder were largely consistent with findings from the Household Pulse Survey during June (*1*).

Markedly elevated prevalences of reported adverse mental and behavioral health conditions associated with the COVID-19 pandemic highlight the broad impact of the pandemic and the need to prevent and treat these conditions. Identification of populations at increased risk for psychological distress and unhealthy coping can inform policies to address health inequity, including increasing access to resources for clinical diagnoses and treatment options. Expanded use of telehealth, an effective means of delivering treatment for mental health conditions, including depression, substance use disorder, and suicidal ideation (*8*), might reduce COVID-19-related mental health consequences. Future studies should identify drivers of adverse mental and behavioral health during the COVID-19 pandemic and whether factors such as social isolation, absence of school structure, unemployment and other financial worries, and various forms of violence (e.g., physical, emotional, mental, or sexual abuse) serve as additional stressors. Community-level intervention and prevention efforts should include strengthening economic supports to reduce financial strain, addressing stress from experienced racial discrimination, promoting social connectedness, and supporting persons at risk for suicide (*9*). Communication strategies should focus on promotion of health services^§§§§^^,^^¶¶¶¶^^,^***** and culturally and linguistically tailored prevention messaging regarding practices to improve emotional well-being. Development and implementation of COVID-19–specific screening instruments for early identification of COVID-19–related TSRD symptoms would allow for early clinical interventions that might prevent progression from acute to chronic TSRDs. To reduce potential harms of increased substance use related to COVID-19, resources, including social support, comprehensive treatment options, and harm reduction services, are essential and should remain accessible. Periodic assessment of mental health, substance use, and suicidal ideation should evaluate the prevalence of psychological distress over time. Addressing mental health disparities and preparing support systems to mitigate mental health consequences as the pandemic evolves will continue to be needed urgently.

**Summary**

**What is already known about this topic?**

Communities have faced mental health challenges related to COVID-19–associated morbidity, mortality, and mitigation activities.

**What is added by this report?**

During June 24–30, 2020, U.S. adults reported considerably elevated adverse mental health conditions associated with COVID-19. Younger adults, racial/ethnic minorities, essential workers, and unpaid adult caregivers reported having experienced disproportionately worse mental health outcomes, increased substance use, and elevated suicidal ideation.

**What are the implications for public health practice?**

The public health response to the COVID-19 pandemic should increase intervention and prevention efforts to address associated mental health conditions. Community-level efforts, including health communication strategies, should prioritize young adults, racial/ethnic minorities, essential workers, and unpaid adult caregivers.

## Footnotes

* https://www.medrxiv.org/content/10.1101/2020.04.22.20076141v1.

† Disorders classified as TSRDs in the *Diagnostic and Statistical Manual of Mental Disorders* (DSM–5) include posttraumatic stress disorder (PTSD), acute stress disorder (ASD), and adjustment disorders (ADs), among others.

§ Unpaid adult caregiver status was self-reported. The definition of an unpaid caregiver for adults was a person who had provided unpaid care to a relative or friend aged ≥18 years to help them take care of themselves at any time in the last 3 months. Examples provided included helping with personal needs, household chores, health care tasks, managing a person’s finances, taking them to a doctor’s appointment, arranging for outside services, and visiting regularly to see how they are doing.

¶ Essential worker status was self-reported. The comparison was between employed respondents (n = 3,431) who identified as essential versus nonessential. For this analysis, students who were not separately employed as essential workers were considered nonessential workers.

** A minimum age of 18 years and residence within the United States as of April 2–8, 2020, were required for eligibility for the longitudinal cohort to complete a survey during June 24–30, 2020. Residence was reassessed during June 24–30, 2020, and one respondent who had moved from the United States was excluded from the analysis. A minimum age of 18 years and residence within the United States were required for eligibility for newly recruited respondents included in the cross-sectional analysis. For both the longitudinal cohort and newly recruited respondents, respondents were required to provide informed consent before enrollment into the study. All surveys underwent data quality screening procedures including algorithmic and keystroke analysis for attention patterns, click-through behavior, duplicate responses, machine responses, and inattentiveness. Country-specific geolocation verification via IP address mapping was used to ensure respondents were from the United States. Respondents who failed an attention or speed check, along with any responses identified by the data-scrubbing algorithms, were excluded from analysis.

†† The surveys contained 101 items for first-time respondents and 86 items for respondents who also participated in later surveys, with the 15 additional items for first-time respondents consisting of questions on demographics. The survey instruments included a combination of individual questions, validated questionnaires, and COVID-19-specific questionnaires, which were used to assess respondent attitudes, behaviors, and beliefs related to COVID-19 and its mitigation, as well as the social and behavioral health impacts of the COVID-19 pandemic.

§§ https://www.qualtrics.com/.

¶¶ Survey weighting was implemented according to the 2010 U.S. Census with respondents who reported gender, age, and race/ethnicity. Respondents who reported a gender of “Other,” or who did not report race/ethnicity were assigned a weight of one.

*** Symptoms of anxiety disorder and depressive disorder were assessed via the four-item Patient Health Questionnaire (PHQ-4). Those who scored ≥3 out of 6 on the Generalized Anxiety Disorder (GAD-2) and Patient Health Questionnaire (PHQ-2) subscales were considered symptomatic for these respective disorders. This instrument was included in the April, May, and June surveys.

††† Symptoms of a TSRD attributed to the COVID-19 pandemic were assessed via the six-item Impact of Event Scale (IES-6) to screen for overlapping symptoms of PTSD, ASD, and ADs. For this survey, the COVID-19 pandemic was specified as the traumatic exposure to record peri- and posttraumatic symptoms associated with the range of stressors introduced by the COVID-19 pandemic. Those who scored ≥1.75 out of 4 were considered symptomatic. This instrument was included in the May and June surveys only.

§§§ For this survey, substance use was defined as use of “alcohol, legal or illegal drugs, or prescriptions drugs that are taken in a way not recommended by your doctor.” Questions regarding substance use and suicidal ideation were included in the May and June surveys only. Participants were informed that responses were deidentified and that direct support could not be provided to those who reported substance use behavior or suicidal ideation. Regarding substance use, respondents were provided the following: “This survey is anonymous so we cannot provide direct support. If you would like crisis support please contact the Substance Abuse and Mental Health Services Administration National Helpline, 1-800-662-HELP (4357), (also known as the Treatment Referral Routing Service) or TTY: 1-800-487-4889. This is a confidential, free, 24-hour-a-day, 365-day-a-year, information service, in English and Spanish, for persons and family members facing mental and/or substance use disorders.” Regarding suicidal ideation, respondents were provided the following: “This survey is anonymous so we cannot provide direct support. If you would like crisis support please contact the National Suicide Prevention Lifeline, 1-800-273-TALK (8255, or chat line) for help for themselves or others.”

¶¶¶ Rural-urban classification was determined by using self-reported ZIP codes according to the Federal Office of Rural Health Policy definition of rurality. https://www.hrsa.gov/rural-health/about-us/definition/datafiles.html.

**** Odds of incidence was defined as the odds of the presence of an adverse mental or behavioral health outcome reported during a later survey after previously having reported the absence of that outcome (e.g., having reported symptoms of anxiety disorder during June 24–30, 2020, after not having reported symptoms of anxiety disorder during April 2–8, 2020).

†††† Adjusted for gender, employment status, and essential worker status or unpaid adult caregiver status.

§§§§ Disaster Distress Helpline (https://www.samhsa.gov/disaster-preparedness): 1-800-985-5990 (press 2 for Spanish), or text TalkWithUs for English or Hablanos for Spanish to 66746. Spanish speakers from Puerto Rico can text Hablanos to 1-787-339-2663.

¶¶¶¶ Substance Abuse and Mental Health Services Administration National Helpline (also known as the Treatment Referral Routing Service) for persons and families facing mental disorders, substance use disorders, or both: https://www.samhsa.gov/find-help/national-helpline, 1-800-662-HELP, or TTY 1-800-487-4889.

***** National Suicide Prevention Lifeline (https://suicidepreventionlifeline.org/): 1-800-273-TALK for English, 1-888-628-9454 for Spanish, or Lifeline Crisis Chat (https://suicidepreventionlifeline.org/chat/).

## References

[1] CDC, National Center for Health Statistics. Indicators of anxiety or depression based on reported frequency of symptoms during the last 7 days. Household Pulse Survey. Atlanta, GA: US Department of Health and Human Services, CDC, National Center for Health Statistics; 2020. https://www.cdc.gov/nchs/covid19/pulse/mental-health.htm

[2] CDC, National Center for Health Statistics. Early release of selected mental health estimates based on data from the January–June 2019 National Health Interview Survey. Atlanta, GA: US Department of Health and Human Services, CDC, National Center for Health Statistics; 2020. https://www.cdc.gov/nchs/data/nhis/earlyrelease/ERmentalhealth-508.pdf

[3] Czeisler MÉ, Tynan MA, Howard ME, Public attitudes, behaviors, and beliefs related to COVID-19, stay-at-home orders, nonessential business closures, and public health guidance—United States, New York City, and Los Angeles, May 5–12, 2020. MMWR Morb Mortal Wkly Rep 2020;69:751–8. 10.15585/mmwr.mm6924e1 32555138 PMC7302477

[4] Löwe B, Wahl I, Rose M, A 4-item measure of depression and anxiety: validation and standardization of the Patient Health Questionnaire-4 (PHQ-4) in the general population. J Affect Disord 2010;122:86–95. 10.1016/j.jad.2009.06.019 19616305

[5] Hosey MM, Leoutsakos JS, Li X, Screening for posttraumatic stress disorder in ARDS survivors: validation of the Impact of Event Scale-6 (IES-6). Crit Care 2019;23:276. 10.1186/s13054-019-2553-z 31391069 PMC6686474

[6] Substance Abuse and Mental Health Services Administration. Key substance use and mental health indicators in the United States: results from the 2018 National Survey on Drug Use and Health. Rockville, MD: US Department of Health and Human Services, Substance Abuse and Mental Health Services Administration; 2018. https://www.samhsa.gov/data/sites/default/files/cbhsq-reports/NSDUHNationalFindingsReport2018/NSDUHNationalFindingsReport2018.pdf

[7] Horesh D, Brown AD. Traumatic stress in the age of COVID-19: call to close critical gaps and adapt to new realities. Psychol Trauma 2020;12:331–5. 10.1037/tra0000592 32271070

[8] Hailey D, Roine R, Ohinmaa A. The effectiveness of telemental health applications: a review. Can J Psychiatry 2008;53:769–78. 10.1177/070674370805301109 19087471

[9] Stone D, Holland K, Bartholow B, Crosby A, Davis S, Wilkins N. Preventing suicide: a technical package of policy, programs, and practices. Atlanta, GA: US Department of Health and Human Services, CDC, National Center for Injury Prevention and Control; 2017. https://www.cdc.gov/violenceprevention/pdf/suicideTechnicalPackage.pdf

